# LC-MS Analysis Revealed the Significantly Different Metabolic Profiles in Spent Culture Media of Human Embryos with Distinct Morphology, Karyotype and Implantation Outcomes

**DOI:** 10.3390/ijms23052706

**Published:** 2022-02-28

**Authors:** Chupalav Eldarov, Alina Gamisonia, Vitaliy Chagovets, Luiza Ibragimova, Svetlana Yarigina, Veronika Smolnikova, Elena Kalinina, Nataliya Makarova, Victor Zgoda, Gennady Sukhikh, Mikhail Bobrov

**Affiliations:** 1 National Medical Research Center for Obstetrics, Gynecology and Perinatology Named after Academician V.I. Kulakov, 4 Oparina Str., 117997 Moscow, Russia; chupalav@gmail.com (C.E.); alyafonya@mail.ru (A.G.); vvchagovets@gmail.com (V.C.); ibragimova_luisa0693@mail.ru (L.I.); s.a.iarygina@yandex.ru (S.Y.); v_smolnikova@oparina4.ru (V.S.); e_kalinina@oparina4.ru (E.K.); np_makarova@oparina4.ru (N.M.); g_sukhikh@oparina4.ru (G.S.); 2 Orekhovich Institute of Biomedical Chemistry, 10 Pogodinskaya Str., 119121 Moscow, Russia; victor.zgoda@gmail.com; 3 Department of Obstetrics, Gynecology, Perinatology and Reproductology, Institute of Professional Education, Federal State Autonomous Educational Institution of Higher Education I.M. Sechenov First Moscow State Medical University of the Ministry of Health of the Russian Federation (Sechenov University), 8/2 Trubetskaya Str., 119991 Moscow, Russia

**Keywords:** exometabolomics, metabolomics, implantation, human embryo, aneuploidy, LC-MS

## Abstract

In this study we evaluated possible differences in metabolomic profiles of spent embryo culture media (SECM) of human embryos with distinct morphology, karyotype, and implantation outcomes. A total of 153 samples from embryos of patients undergoing in vitro fertilization (IVF) programs were collected and analyzed by HPLC-MS. Metabolomic profiling and statistical analysis revealed clear clustering of day five SECM from embryos with different morphological classes and karyotype. Profiling of day five SECM from embryos with different implantation outcomes showed 241 significantly changed molecular ions in SECM of successfully implanted embryos. Separate analysis of paired SECM samples on days three and five revealed 46 and 29 molecular signatures respectively, significantly differing in culture media of embryos with a successful outcome. Pathway enrichment analysis suggests certain amino acids, vitamins, and lipid metabolic pathways to be crucial for embryo implantation. Differences between embryos with distinct implantation potential are detectable on the third and fifth day of cultivation that may allow the application of culture medium analysis in different transfer protocols for both fresh and cryopreserved embryos. A combination of traditional morphological criteria with metabolic profiling of SECM may increase implantation rates in assisted reproductive technology programs as well as improve our knowledge of the human embryo metabolism in the early stages of development.

## 1. Introduction

The application of the assisted reproductive technologies (ART) has been constantly improving over the last four decades since the first IVF-assisted birth in 1978 [1]. While the number of ART cycles steadily increase worldwide, live birth rates vary significantly over different regions—from 12–51% for fresh embryos to 18–57% for cryopreserved ones [2].

One of the ways to increase ART efficiency is double embryo transfer [3,4,5,6], but this approach has well-known risks of multiple pregnancies, associated with different complications for infant and mother health [7,8], which have tendency to increase with age [9,10]. Over the past two decades, there has been a shift towards the elective single embryo transfer (eSET) protocols [11]. While this approach mitigates risks associated with multi-pregnancy, it requires developing an accurate strategy of embryo quality assessment, selection, and transfer. Initially, the question was raised of when the embryo should be transferred. Centers of Disease Control and Prevention (CDC) in the USA reported that in 2013, most of the embryos were transferred on day 3 at the cleavage stage or day 5 at the blastocyst stage, but transfers on day 1, 2, 4, and 6 have been reported as well [12]. A meta-analysis including 1654 patients has shown significantly higher clinical pregnancy and live birth rates among embryos transferred at the blastocyst stage compared to those transferred at cleavage state. The 5th-day blastocysts have better synchronization with an IVF stimulated endometrium and the high implantation potential [13]. One of the most obvious and widely used methods of embryo quality assessment utilizes this correlation and is based on morphological criteria, which considers blastocoel size, form, and shape of trophectoderm and inner cell mass for the evaluation of embryo development and viability [14]. Currently, the morphology-based grading system of embryo quality assessment is also enhanced by morphokinetics analysis using time-lapse light microscopy [15,16,17] and deep machine learning methods [18]. However, embryo morphological phenotype evaluation alone is insufficient for accurate prediction of successful implantation in the situation of choice between multiple embryos of “excellent” (“A”) grade [19,20]. Moreover, aneuploid embryos can form morphologically high-scoring blastocysts or even reach the term of delivery. Despite the existing correlation between karyotype and morphology [21], classic grading or time-lapse microscopy is not an alternative to genetic analysis for accurate assessment of embryo karyotype [22,23,24,25]. At the same time, genetic abnormalities highly interfere with implantation and physiological development [26]. That is why methods of preimplantation genetic testing for aneuploidies (PGT-A) are generally used to check the karyotype of selected embryos before the transfer [27,28,29]. However, this invasive method can result in decrease of pregnancy rates if biopsies are performed at the cleavage stage [30,31]. No such effects were found after blastocysts biopsies, but the technical complexity of PGT-A using NGS or array hybridization makes it applicable only for frozen-thawed embryos to avoid day 6 fresh transfers, which were reported to be associated with lower success rates [32]. The high cost of PGT-A procedures can also be considered as a limiting factor for broad method application.

Limitations of morphology-based and genetic classification methods stimulated the search for new approaches for the non-invasive biochemical or molecular evaluation of embryo viability and quality. One of the promising non-invasive approaches includes measurements of biochemical intermediates, such as glucose, lactate, pyruvate, amino acids, b-HCG, or other proteins in the spent embryo culture medium [33,34,35,36]. Recently, several spectroscopy methods (Raman spectroscopy, NMR spectroscopy, IR spectroscopy) have been used to study the embryonic metabolome [37,38,39] as well as gas chromatography coupled with mass spectrometry [40]. The embryo metabolome might reflect the dynamic changes of cell activity regulated by the expression of certain sets of genes and their target proteins, affecting the molecular phenotype of the embryo and its implantation potential. The metabolic activity of the embryo can be assessed by examining the exometabolome of the spent culture medium, which is formed by dynamic nutrient intake and secretion of metabolites. Thus, exometabolomics of spent embryo culture medium (SECM) might provide useful insights on the functional features of embryonic development and overall embryo viability to improve the success rates of the eSET protocol.

One of the most advanced and powerful methods of metabolites profiling is the combination of high-performance liquid chromatography followed by mass spectrometry (HPLC-MS). This approach is widely approved in modern omics research, but it is still rarely used for human embryo culture media analysis. In this study, the HPLC-MS method was used to evaluate the differences in the metabolomic profiles of culture media from human embryos of different morphological classes and karyotypes. Metabolite content of paired SECM on the 3rd and 5th day of cultivation was also studied to show dynamic changes and check the reproducibility of results on different LC-MS systems. Finally, changes in the metabolomic profiles of SECM from embryos with different implantation outcomes after transfer were analyzed.

## 2. Results

### 2.1. Influence of Morphology and Karyotype on the Composition of Spent Embryo Culture Media

The composition of the metabolites in the culture media may depend both on the total metabolic activity of all the cells of the embryo, and on their number by a certain day of development. To evaluate the influence of the development stage on the embryo exometabolome, SECM samples (*n* = 153) obtained on day 5 of continuous incubation were selected without considering the karyotype. We assumed that the embryos that reached the optimal parameters of development, regardless of the karyotype, would have minor differences in metabolic activity. The number of samples in each class and their morphological grades are shown in Table 1. The distribution of embryos by classes was based on morphological criteria and the probability of their transfer. Only blastocysts with expansion score ≥3 according to Gardner classification were considered for the analysis. That is, first-class embryos always have priority for transfer, second-class embryos are transferred when first-class embryos are absent or when they show features of an aneuploid karyotype. Third-class embryos usually show development retardation signs and are generally not recommended for transfer. Finally, the fourth class includes embryos that stopped developing at the stage of one to several cells and can be considered as a negative control. Embryos in morulae stage having smaller number of cells and blastocysts with the 1 and 2 expansion grades were excluded due to low probability of clinical pregnancy and lower frequency of their transfer.

**Figure 1:**
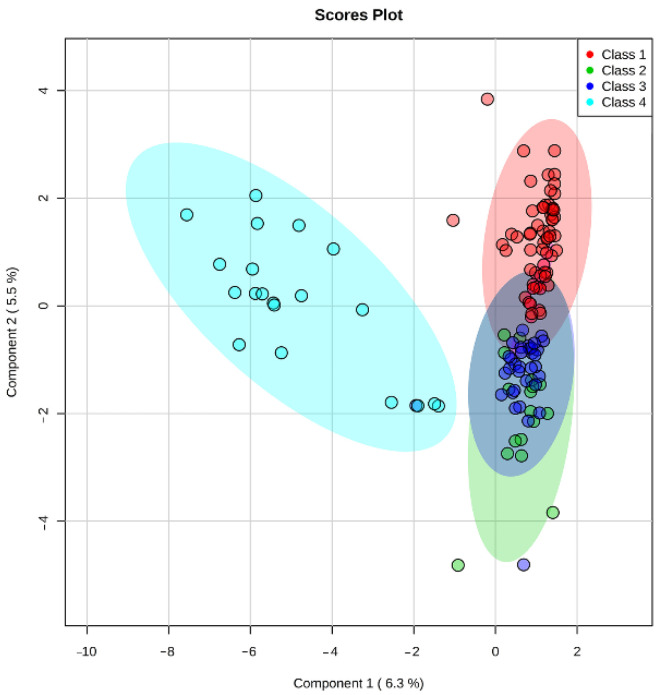
Score plot of intergroup PLS-DA of day 5 culture medium samples from embryos of different morphological classes.

After processing the HPLC-MS data, 1046 ions were detected in the mass range *m*/*z* 100–804. Multivariate statistics methods are widely used to calculate and visualize variation of thousands of features found by untargeted metabolomics, resulting in fast sample clusterization and outlier filtering. To visualize the difference in metabolic profiles of the samples by morphological class, partial least-squares discriminant analysis (PLS-DA) was applied to the HPLC-MS data. PLS-DA showed a clear clustering of nutrient media samples from different morphological classes. Class 4, used as control, significantly differed from the other three classes, which may indicate a notable contribution to the changes in the composition of the nutrient media by actively developing embryos. It can also be seen that Class 1 tends to cluster away from the “good” and “fair” embryos from Classes 2 and 3, respectively (Figure 1).

**Figure 2:**
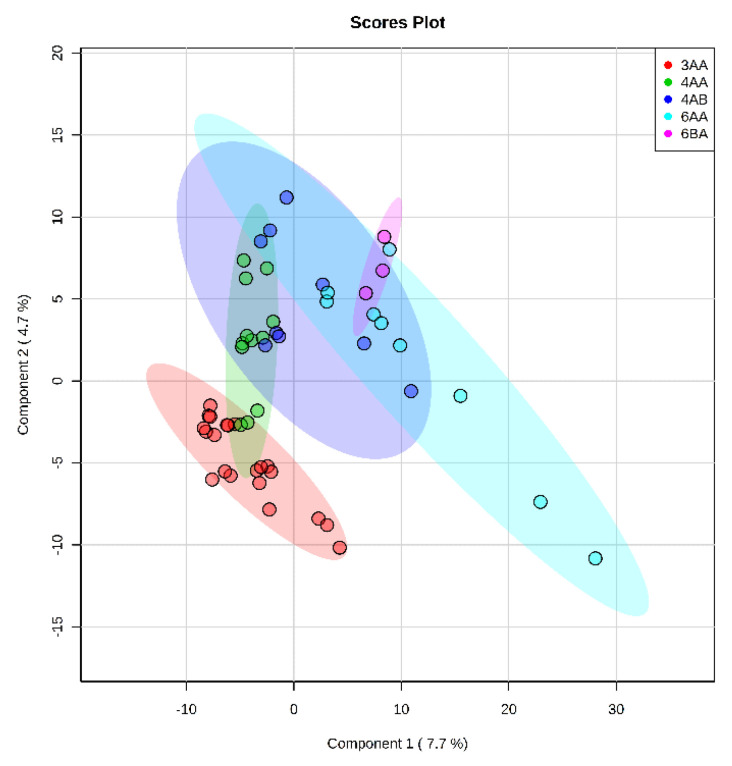
Score plot of intragroup comparison of metabolite profiles of day 5 culture medium samples from the class 1 embryos by PLS-DA. Samples are grouped according to morphological classification for day 5 embryos.

To assess possible differences within the same class, a PLS-DA analysis was performed between embryos with different morphology in Class 1: 6AA, 6BA, 4AA, 4AB, 3AA (Figure 2). Grades 3AA having fewer cell numbers showed minor differences and tended to separate from the rest of the embryo samples, but no clear clustering within the class was observed. Thus, the different morphological classes of embryos showed class-specific features that distinguish the groups from each other, suggesting that the exometabolome profile of the blastocyst correlates with cell number and the stage of development. Although we observed the tendency of Classes 1–3 to separate, for further steps of the study we selected samples of only fully developed blastocysts of 3–6 AA grades from Class 1.

**Figure 3:**
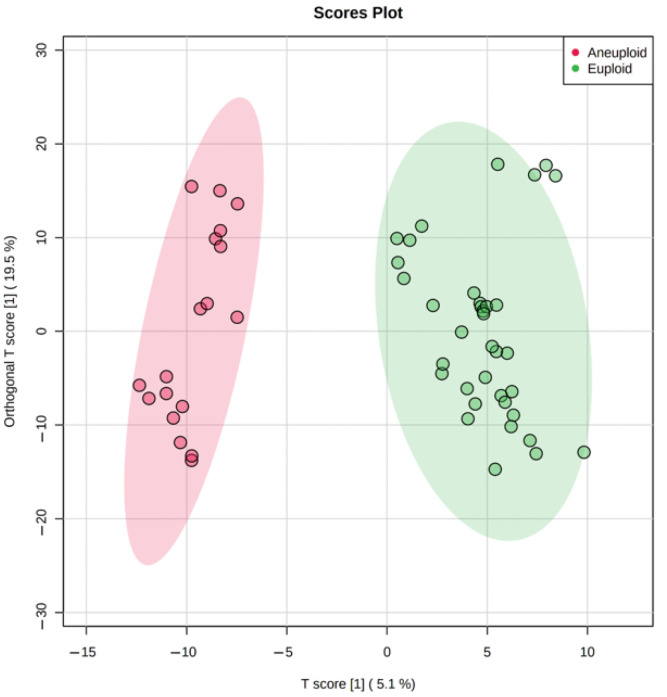
Score plot of OPLS-DA comparative analysis of metabolomic profiles of day 5 culture media samples from the 1st class embryos with euploid and aneuploid karyotypes.

One of the most important criteria for selecting an embryo for transfer is the quality of its karyotype. In our study, a rather high incidence of karyotype abnormalities was detected by PGT-A: 12 of the 35 selected embryos from Class 1 and 21 of the 43 from Class 2 (Table 1). Among aneuploid embryos from both classes, twenty had one abnormality, eight had two, and five had three or more abnormalities (mono- or trisomies). To assess the influence of the altered karyotype on the metabolite profile, samples of nutrient media from aneuploid and euploid embryos of the first morphological class were analyzed. The score graph of the multivariate OPLS-DA revealed a good clustering of samples by karyotype (Figure 3). This fact indicates that the detected aneuploidies may affect the metabolic activity of embryos, despite the “normal” morphological phenotype.

To detect the molecular signatures that contribute the most to the differences between the euploid and aneuploid groups, molecular ions with a fold change of ≥2 were filtered by univariate statistics (*p* < 0.05) and mapped to Human Metabolome Database (HMDB, http://www.hmdb.ca/. Accessed on 20 January 2022). Among 23 molecular ions selected by such criteria, only two were identified by HMDB (Table 2). Their possible structures refer to: 16-hydroxy-10-oxohexadecanoic acid (*m*/*z* 309.2042) and 5,6-dihydroxyprostaglandin F1 (*m*/*z* 389.2517). Despite the clustering of the samples by the OPLS-DA method, the univariate statistical analysis with used parameters did not show any significant differences in common, easily identifiable components of the medium, such as amino acids. The content of most of the detected metabolites (22 out of 23) was increased in the culture media of aneuploid embryos, which indicates a possible metabolic shift in this group (Table 2). To further investigate the differences between groups, all molecular ions marked as significant for the OPLS-DA model were also mapped to HMDB, and their pre-identification within 15 ppm (Table S1) was used to enrich the KEGG pathways. The results of the pathway enrichment are presented in Table 3. It was shown that most of the enriched pathways relate to the amino acids, fatty acids, vitamins B and A metabolism. The most pronounced topological impact was shown for vitamin B6 metabolism and pentose phosphate pathway.

### 2.2. Implantation Potential

The excellent morphology and normal karyotype of the embryo do not guarantee its successful implantation, which further complicates the choice of the embryo for transfer. To study the possible correlation between the implantation potential of the transferred embryos and the composition of their nutrient media, a retrospective analysis of samples from euploid embryos with known transfer outcomes was performed. Based on the data obtained at the previous stage, morphologically similar embryos of the excellent class were selected.

**Figure 4:**
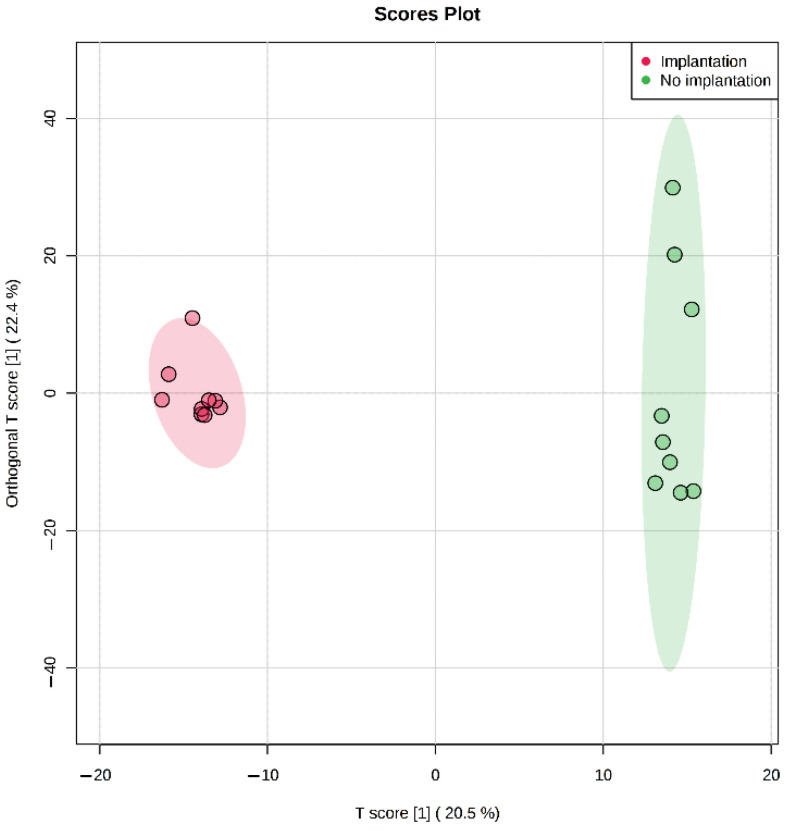
Score plot of OPLS-DA comparative multivariate statistical analysis of metabolomic profiles of day 5 culture media samples from embryos of the first morphological class with successful and unsuccessful implantation.

In SECM from embryos continuously cultured for 5 days multivariate statistical analysis (OPLS-DA) revealed significant differences between the groups with the corresponding transfer outcomes, which indicates an essential role of the embryo’s metabolic rate in culture for subsequent successful implantation (Figure 4). Among the detected molecular ions, 241 were found to have significant (*p* < 0.05) fold change ≥2 in groups with different implantation potential (Table S2). To further search for possible implantation-specific signatures, molecular ions with a statistically significant fold change (≥2) between the groups were selected and mapped to the HMDB for identification. From 241 ions, only 100 had HMDB IDs giving 1214 different molecular identifications, of which only 142 had KEGG IDs indicating their possible involvement in characterized metabolic pathways (Table S2). Several selected ions of interest are shown in Table 4.

**Figure 5:**
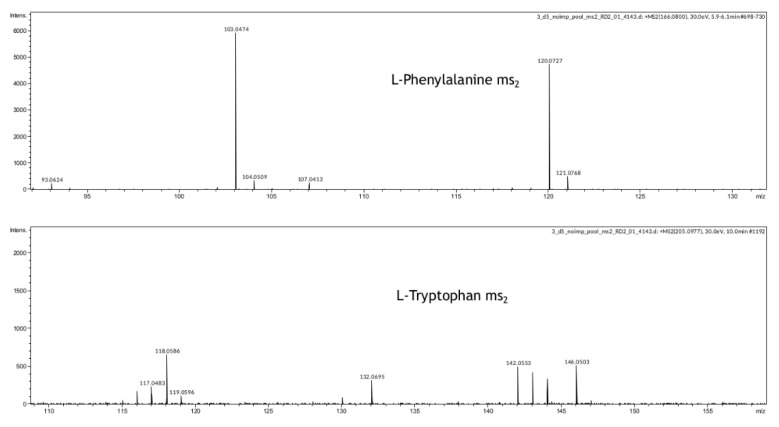
Mass spectra of MSn analysis for L-Phenylalanine and L-tryptophan.

The known constituents of embryonic culture medium are amino acids, which may be used in synthetic and energetic metabolism. The pronounced decrease was observed in the relative abundance of molecular ions identified as phenylalanine following tryptophan, valine, and proline. HMDB identification was validated by comparing retention times of found amino acids with ^13^C^15^N-labeled amino-acid standards (Table S3, Figure S1). The most abundant molecular ions were fragmented for structural identification in MSn experiment and their initial identifications were confirmed (Figure 5). However, MSn analysis was limited by low absolute abundance of the most of active SECM components due to small initial sample volume. All molecular ions marked as significant for the OPLS-DA model were also mapped to HMDB, and their possible identification within 10 ppm of *m*/*z* was used to enrich the KEGG pathways. It was found that the most enriched pathways relate to the biosynthesis and metabolism of amino acids phenylalanine, valine, arginine, and several others (Table 5), pathways involved in lipid and nucleotide metabolism were also revealed (Table 5).

**Figure 6:**
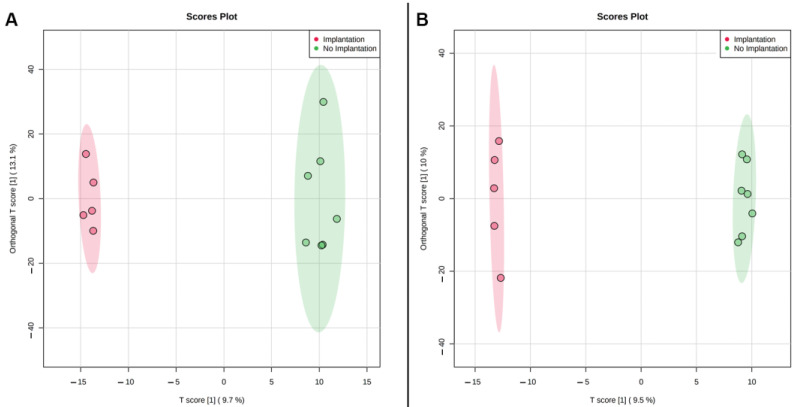
Score plot of OPLS-DA of metabolomic profiles of day 3 (**A**) and day 5 (**B**) culture media samples from euploid embryos of 1st morphological class with successful and unsuccessful implantation.

In discontinuous protocol HPLC-MS analysis of SECM samples of the third and fifth days revealed 1616 and 1404 molecular ions in the mass range *m*/*z* 86–804, respectively. These paired SECM samples were divided into two groups depending on the outcome of embryo implantation: successful (*n* = 5) or unsuccessful (*n* = 7). The data were subjected to multivariate statistical analysis using OPLS-DA to visualize clustering and find the ions that contribute most to the variance between the two groups. Explicit clustering of the two groups corresponding to different implantation outcomes was detected on both the third and fifth day of cultivation (Figure 6A,B). Molecular ions with a statistically significant fold change (≥2) between the groups were selected: 50 (17 decreased, 34 increased) and 36 (21 decreased, 15 increased) molecular ions on the third and fifth day, respectively (Table S4). In addition, the ions significant for the OPLS-DA model were mapped to HMDB, and the potential metabolites were enriched with the KEGG database (Table 6 and Table 7). Several amino acids, lipid and purine metabolism pathways were enriched both at day 3 and day 5 of cultivation. Interestingly, phenylalanine-associated pathways were enriched in day 5 SECM in both protocols but were absent in day 3 SECM of the discontinuous protocol. Retinol metabolism pathway was enriched in day 3 and 5 SECM of the discontinuous protocol, while in continuous protocol this pathway did not contribute to variation between groups of SECM with successful and unsuccessful implantation (Table 6 and Table 7).

### 2.3. Blank Embryo Culture Media Decay Analysis

To investigate possible changes of culture media composition over time and their effect on results, we cultivated blank culture media for five days and compared it with a fresh one. More than 650 molecular ions were initially detected by LC-MS analysis and groups (fresh and day 5 media) showed excellent clusterization by OPLS-DA, but only 60 molecular ions were found to change with statistical significance at *p* < 0.05 (with FDR correction). We compared these ions with molecular ions contributing to difference between groups with different implantation outcome to detect any batch effect. Only 9 matching molecular ions were found (Table S5). We used HMDB database to identify these molecular ions and found no interference with our results data. Thus, possible natural embryo media decay over 5 days of cultivation does not have any significant impact on results and is negligible.

## 3. Discussion

The elective single-embryo transfer has been shown in several studies to be the recommended method compared to multiple embryo transfer, since it demonstrates a similar birth rate and is devoid of disadvantages associated with multiple pregnancies [41,42,43]. However, this method requires a comprehensive assessment of the viability and implantation potential of a single embryo, as there is no room for error. It should be noted that currently there are no reliable criteria for choosing the best candidate from two euploid embryos with excellent morphological qualities. To address these issues, routine morphology analysis, even with time-lapse microscopy, should be supported by biochemical or molecular methods. One of such methods is metabolomic profiling which allows taking of a “snapshot” of current metabolites composition which might correlate with the embryo functional condition and implantation potential. While direct measurement of embryo metabolism is complicated, spent culture media exometabolome may provide crucial information on embryo development based on different nutrient consumption and metabolites excretion to media by embryos of different morphological quality or the same quality but different implantation potential.

In this study, metabolite profiles of spent culture media were obtained in two cultivation protocols: five days of continuous cultivation or five days of discontinuous cultivation with a complete refreshment of the media on day 3. The media samples were grouped into four classes according to embryo morphological grade and their quality for transfer (Table 1). This classification was based on the experience of ART specialists of our clinic and literature data. Gardner et al. showed that embryos with ≥3AA grades had the highest probability to give clinical pregnancy [44]. Additionally, in a recent article Zhao et al. 2018, based on Gardner’s classification, divided the embryos according to morphological characteristics into four classes: excellent (3–6, AA), good (3–6 AB, BA), medium (3–6 BB, AC, CA), poor (1–6, combinations of B and C). It was shown that embryos of excellent and good quality have a close probability of clinical pregnancy (65 and 59%, respectively), while in the middle and poor classes these parameters decreased (50 and 33%, respectively) [45]. In our study, we assigned some of the embryos of good quality (mentioned above) to an excellent class. We assumed that embryos with similar efficiency should have similar metabolic features; however, the number of cells in the embryo may also affect the quantitative parameters of metabolites in culture media. Unfortunately, the exact number of cells in each morphological group of human embryos is unknown (at least we could not find such data). This is apparently due to the lack of non-invasive evaluation methods and the inability to use many human embryos in experiments. Therefore, we conditionally divided the samples available at the time of the study, as indicated in Table 1.

Multivariate PLS-DA statistics were used for intergroup (Figure 1) and intragroup (Figure 2) comparative analysis. Intergroup PLS-DA for the samples obtained in continuous protocol showed clustering of ‘excellent’ (Class 1) and ‘poor’ (Class 4) groups of embryos from ‘good’ (Class 2) and ‘fair’ (Class 3) groups that were mostly overlapped. It can also be seen that Class 1 and Class 3 tend to separate while Class 2 overlaps with them. A possible explanation is that Class 2 includes groups of embryos that have a similar number of cells to both Class 1 and Class 3. Since the morphological evaluation by the embryologist does not allow for an accurate cell count of each embryo, this circumstance may also contribute to the observed intersections of clusters of similar morphological groups. This result might indicate that a correlation exists between the embryo’s developmental stage and its metabolic activity. Class 1 embryo is expected to have normal phenotype and thus have optimal metabolic activity, and metabolic rates of Class 2 and 3 embryos may be variable due to different possible origins of their “fair” phenotype and lower cell number, while Class 4 embryo should have the lowest metabolic rates due to significant retardation or the arrest of the development. It may be proposed that in embryos with the smaller cell numbers there is a shift in gene expression leading to the formation of ATP-dependent ion gradients necessary for blastocele formation that takes a large percentage of the embryo’s energy to create and maintain the blastocoel cavity [46]. In these embryos, this process may follow in expense of the others, such as cell division. Hence the embryo must possess some degree of metabolic competency to arrange its full-fledged development.

Intragroup comparison made for Class 1 embryos showed that metabolite profiles within the class are quite similar for different grades (Figure 2). One possible explanation may be that any significant metabolic profile shift would significantly influence the development with a consequent class change. Thus, in Class 1, minor variability is observed, with a slight clustering of type 3AA embryos, which may be associated with a smaller number of cells in this group. Additionally, in 3AA embryos, the metabolic pathways responsible for expansion are just beginning to activate compared to more developed ones. Detected differences between morphological groups indicate the need for careful selection of samples according to the specified criteria since they can have a significant impact on the results of the metabolomic analysis.

The metabolomic profiling of SECM has been done previously by a range of spectroscopic methods including Fourier Transform infrared spectroscopy (FT-IR), near-infrared spectroscopy (NIR), Raman spectroscopy [47,48] and NMR [39]. These studies showed differences in metabolism ratios which correlating with embryo viability and implantation potential [39,49,50,51,52]. Seli et al. used NIR spectroscopy and found that viability indices calculated on metabolic profiles of day 2–3 SECM are independent of embryo morphology and correlate with reproductive potential of embryos [53]. This work received considerable interest and discussion. Subsequent randomized control studies of spent embryo culture media on day 2 and day 5 by NIR spectroscopy in adjunct to morphology did not confirm any significant improvement of ongoing pregnancy rates compared to classic morphology-based criteria [54]. Another randomized control study of day 3 SECM with NIR spectroscopy also failed to find any improvement in transfer outcome by this combined approach [55]. Evaluation of the technical design of the experiment revealed that such results were obtained due to limitations of the existing equipment. Additionally, the signal-to-noise ratio threshold was not strong enough for correct viability predictions and noise filtration, which impaired method robustness and reproducibility [56,57].

Raman spectroscopy, while being a powerful non-destructive analysis method with high specificity, has relatively low sensitivity, which can limit its usage for analysis of complex biological fluids, such as serum plasma or spent culture media [58]. Nutrient consumption and metabolite excretion rates even between excellent, fully developed blastocyst and poor-quality morula can differ by a narrow margin, which requires highly sensitive analysis methods. Some highly active compounds, such as hormones or mediators, usually have extremely small concentrations, and their detection is also complicated. Mass-spectrometry coupled with gas or liquid chromatography is an effective and versatile method of analysis, that provides an alternative for spectroscopy-based metabolomic profiling. Cortezzi et al. used direct MS analysis, without chromatographic separation, of the day 3 culture medium with known implantation outcome to predict their implantation potential. The spectra were obtained in negative-ion mode and analyzed by PLS-DA to test the performance of the statistical model on implantation potential prediction. The model correctly identified 100% of positive implantations and 70% of negative ones. The culture media from embryos with positive or negative outcome showed specific biochemical signatures which contribute the most to the difference between groups, though no identification was provided for the detected ions [59]. While untargeted metabolomics of spent culture media is good for biomarkers search, there is often a need not to discover new, but thoroughly identify and quantify existing biomarkers found by biochemical or molecular methods. Targeted free fatty acid LC-MS profiling revealed a statistically significant decrease of docosahexaenoic acid and an insignificant decrease of other essential free fatty acids in day 6 spent culture from morphologically “good” embryos compared to “poor” ones. Authors suggested targeted metabolomic profiling as a powerful method for non-invasive assessment of embryo quality [60].

The genetic analysis revealed a substantial amount of Class 1 and 2 embryos with an abnormal karyotype (Table 1), hence we tested metabolomic profiling as a possible non-invasive alternative for PGT-A. Multivariate statistics (OPLS-DA) has divided culture media samples of the aneuploid group from the normal group within Class 1 (Figure 3), demonstrating the potential influence of the karyotype on metabolite content. Univariate statistics revealed 23 molecular ions differing between these groups (the fold change > 2). Interestingly, 22 of 23 had increased abundance, probably due to decreased uptake of medium components. Most of these 23 ions were not identified by HMDB search within 10 ppm mass accuracy. It can be assumed that with significant changes in the metabolism of aneuploid embryos, they would not have reached the appropriate stage of development and would not have shown the best phenotype. This may explain the fact that we were not able to detect pronounced changes among the known components of the nutrient media and their possible metabolites. However, multivariate statistics have revealed sets of ions, the cumulative change in the content of which has led to the clustering of groups depending on the karyotype. This suggests that aneuploid embryos may have specific differences in metabolism, and certain molecular signatures can be used to detect aneuploidies.

For the evaluation of the overall metabolic activity of embryos with different karyotypes, all significantly different molecular ions defined by multivariate statistics were mapped to the HMDB database, and metabolites identified were enriched by KEGG biochemical pathways database. Amino acids and fatty acids metabolism pathways were among the most enriched ones, which is fully in accordance with the known importance of amino acid turnover for normal embryo development [61]. Aneuploidies are known to have a devastating effect on embryonal development, affecting all levels of the organization, and only rare karyotype abnormalities like trisomy of chromosome 21 are tolerable enough to give live birth but associated with multiple organ pathologies and disorders [62,63].

Due to the incident nature of the karyotype abnormalities, we combined all types of aneuploidies including monosomy, trisomy, and their combinations in one group and analyzed them against euploid embryos. However, the multivariate statistical analysis still shows excellent clustering of euploid and aneuploid embryos, though the contribution of single metabolites might have been smoothed by sample heterogeneity (Figure 3). Further studies with samples grouping by individual types of aneuploidies will likely reveal specific features in the composition of metabolites that are characteristic of each karyotype abnormality. Research conducted by Sanchez-Ribas et al. confirms this assumption [36]. It was shown that several potential metabolites can distinguish trisomy 21 embryos from normal ones although authors had to group ‘pure’ trisomy 21 samples with those having trisomy 21 in combination with other aneuploidies due to extremely rare occurrence of ‘pure’ trisomy 21 [36]. The preliminary results from another study suggest that metabolomics markers may be sensitive to trisomy 18 and may be able to distinguish trisomy 18 from trisomy 21 [64]. Raman spectroscopy and multivariate statistics were recently used to detect aneuploid embryos. The method showed results consistent with PGT-A testing performed by next-generation sequencing, achieving 95.9% of correct detections of either euploidy or aneuploidy [65]. Significant differences in Raman spectra associated with small RNA and lipids were also observed. Thus, metabolomic profiling coupled with targeted metabolomics could be a feasible method for the non-invasive detection of aneuploidies.

In our study, we have also demonstrated that metabolomic profiling of spent culture media of day 5 embryos from the same morphological class can be used to assess their implantation potential. After five days of incubation molecular ions corresponding to phenylalanine, valine, proline, and tryptophan were found to be decreased in spent culture media from embryos with successful implantation, changes of other amino acid derivatives were observed as well (Table 4 and Table S3). Proline, valine, and phenylalanine identifications were confirmed using isotope-labeled standards (Table S3, Figure S1), phenylalanine and tryptophan were further confirmed by MSn analysis (Figure 5). This result is in accordance with previous reports of the amino acid turnover rate as a predictor of embryo viability [34]. It was also shown that differences in the composition of the culture media of the implanted embryos (Class 1) can be detected on the 3rd day of cultivation, as well as on the 5th day after media replacement. In spent culture media of days 3 and 5, 46 and 29 statistically differing molecular ions were found by univariate statistics. No amino acids were identified among the changing features except glutamine. That may be explained by the fact that culture media was refreshed on day 3 in this study, which might negate all accumulated composition differences observed after 5 days of cultivation in continuous protocol. It can be concluded that differences between embryos with distinct implantation potential can be seen starting at least from day 3, which may allow the application of culture medium analysis in different transfer protocols for both fresh and cryopreserved embryos.

Pathway enrichment of metabolites marked as important by multivariate statistics was performed for all implantation outcomes and karyotype comparisons. The most enriched pathways for compounds contributing to karyotype differences were related to the pentose phosphate pathway, vitamin B6, and amino acid metabolism pathways. Glucose-dependent nucleotide synthesis by the pentose phosphate pathway was found to control activation pathway for trophectoderm specific gene transcription during mammalian embryogenesis [66]. Amino acids turnover and high levels of vitamins A and B6 were previously reported to be associated with aneuploidies, embryo quality, and morphokinetics [61,67]. The enrichment of amino acid and vitamin B6 metabolism pathways, as well as lipid-associated pathways, were also observed (Table 5, Table 6 and Table 7) in culture media of embryos with successful or unsuccessful implantation. Drabkova et al. showed that there were not any significant changes in media amino acid composition in day 3 SECM. Additionally, positive correlations with glutamine and negative with glutamate concentration were reported [68]. Our findings also show increased glutamine depletion in day 3 medium from embryos successfully implanted later (Table S4). Amino acids are the key components of a variety of pathways, even those not directly involved into amino acid biosynthesis or degradation.

Earlier studies of lipid effects on embryo quality revealed a significant impact of fatty acids levels on embryo development [69,70]. Fatty acid derivatives such as eicosanoids may be produced on the blastocyst–endometrium interface and seem to be one of the key factors of the decidualization and implantation process [71]. Our results indicate that lipid metabolism may play significant role during embryo development and specific lipid compound sets should be investigated as potential biomarkers for karyotype and embryo assessment.

In a recent study, Harden S.L. et al. investigated the exometabolome of decidualizing human stromal cells in culture and concluded that the metabolic footprints of cellular microenvironment generated by different decidual subpopulations encode spatiotemporal information that may be important for their differentiation and optimal embryo implantation. It has been shown that in all investigated timepoints of decidualization the most enriched metabolic pathways of stromal cells exometabolome are phenylalanine, tyrosine and tryptophan biosynthesis, valine, leucine, and isoleucine biosynthesis, glycerophospholipid metabolism, linoleic acid metabolism, purine and pyrimidine metabolism [72]. It is interesting to note, that the similar pathways were enriched after analysis of day 5 human embryo exometabolome in our study. It is intriguing to propose that these pathways and their secreted metabolites are necessary for the embryonal and endometrial cells to form complementary microenvironment that guide the initial steps of the implantation. Together, these findings support the importance of found molecular signatures as possible markers of successful implantation. Thus, metabolomic profiling of spent culture media may be a promising method in combination with morphological analysis as it provides additional information on embryo quality. Even two morphologically identical embryos may have different metabolic activity and thus implantation potential, or class 2 embryo may have worth morphology but far better metabolic rate. This fact may be crucial for successful eSET, especially with increasing age, when the number of available embryos is limited.

It should be noted that not all successful implants develop into a full-fledged pregnancy and result in delivery. This may be due to different factors such as incorrect localization of the implanted embryo, abnormalities of the placenta development, and fetus gestation [73]. Therefore, after embryo transfer long-term monitoring and detailed analysis of pregnancy outcomes are necessary for further method development. Another limitation of the method may be the small volume of the probe, which makes precise identification and quantification of low abundant metabolites a challenging task. The method we used showed good results in aneuploidies detection, but we pulled all aneuploidy types to one group because of the rare occurrence of such cases. A longer collection period and more test samples for each type of aneuploidy are required for method refinement and validation. Additionally, we understand that our data was obtained using one type of the commercial culture media provided on the market for human embryo cultivation, thus any differences in composition and nutrient ratio (which are known to exist and often not evident due to unavailable media formulations) may lead to the specific metabolic shifts that in turn will change features of metabolomic profile. Thus, to obtain the set of “universal” biomarkers of embryo quality it seems reasonable to evaluate the difference between metabolomic profiles according to corresponding culture conditions.

In conclusion, our data suggest that the application of HPLC-MS for metabolomic analysis can be used as a powerful, sensitive, and versatile method that may provide valuable data both for embryo karyotype and implantation potential assessment. Further implementation of targeted metabolomics in combination with time-lapse morphology analysis may significantly improve the positive outcome ratio after embryo transfer.

## 4. Materials and Methods

### 4.1. Oocyte Collection, Embryo Cultivation, and Selection

Ovarian hyperstimulation was performed according to the protocol using gonadotropin and gonadotropin-releasing hormone antagonists. The stimulation protocol was the same for all participants. Follicular maturation was monitored by ultrasound examination and was estimated as sufficient if there were more than 5 follicles of ≥18 mm in diameter. Oocytes were collected by ultrasonography-guided transvaginal aspiration of the follicles. Oocyte fertilization was performed by intracytoplasmic sperm injection (ICSI). Human embryos were cultivated in 30 µL droplets of Continuous Single Culture medium (FUJIFILM Irvine Scientific, Santa Ana, CA, USA) for 3–5 days under mineral oil (FUJIFILM Irvine Scientific, Santa Ana, CA, USA) at 37 °C in a gas atmosphere of 89% N_2_, 5% O_2_, and 6% CO_2_. Assisted hatching was performed on the 5th day of cultivation, after the evaluation of embryo morphology by the expert embryologist. Embryos of different morphological quality were marked by alphanumerical indices according to the Gardner grade system [14]. The numbers indicate the expansion grade of a blastocyst (1–4 blastocoel cavity development, 5–6 hatching out of the shell), the first letter indicates intracellular mass quality, and the second letter is for trophectoderm layer quality. The letters have the following meaning: A—many densely packed cells, B—fewer cells, loose packing, C—just a few cells. Embryos were grouped into four arbitrary classes: first “Excellent”, second “Good”, third “Fair”, fourth “Poor”. The distribution of morphological grades between classes is presented in Table 1.

The karyotype evaluation was performed for the selected embryos of the 1st and 2nd classes, which are the most likely to be transferred during the single embryo transfer protocol. Blastocyst trophectoderm biopsy was performed for the preimplantation genetic testing for aneuploidy (PGT-A), and karyotype quality was assessed by array comparative genomic hybridization [74]). After the biopsy, day 5 embryos were cryopreserved and stored until transfer. After two menstrual cycles, according to the results of PGT, cryopreserved/thawed euploid embryos were transferred into the uterine cavity with the preimplantation support using micronized progesterone. Data from only the first transfer were considered for patients undergoing more than one embryo transfer, and only one cycle of oocyte donation was included in case of multiple retrievals. Clinical pregnancy after the transfer was defined by the positive hCG test two weeks after the transfer and ultrasound confirmation by visualization of the gestational sac on third week after embryo transfer and at 5–6 weeks to detect the heartbeat.

### 4.2. Metabolomic Profiling

#### 4.2.1. Samples

Spent embryonic culture media samples (*n* = 153) obtained on day 5 of continuous incubation were selected for the study, of which 35, 43, 20, and 55 belonged to the 1st, 2nd, 3rd, and 4th morphological quality classes, respectively. In discontinuous protocol, SECM samples were collected on day 3, and cultivation was continued in fresh media till day 5. Paired SECM samples (*n* = 24) were obtained on day 3 and day 5 from 12 “excellent” quality embryos. Equal volumes (20 μL) of spent culture media were taken for the experiment, labeled, and stored at −80 °C. To assess possible changes in media composition during the cultivation period additional 6 control samples of blank embryo culture media were analyzed on day 3 and day 5 of incubation.

#### 4.2.2. Sample Preparation

Methanol, acetonitrile, and formic acid (all are HPLC grade) were purchased from Merck (Darmstadt, Germany). Deionized water was prepared with a Milli-Q Water Purification System (Darmstadt, Germany). Samples for further HPLC-MS analysis were prepared by adding 60 µL of methanol to 20 µL of SECM, followed by mixing and centrifugation at 14,000× *g*. The resulting supernatants were aliquoted and kept at −80 °C until the analysis. Mix of 17 ^13^C^15^N-labeled amino acids (Cambridge isotope, Cambridge, UK) was added to supernatants with final concentration of 25 mM as internal standard. The same mix in final concentration of 31.25 mM was used as QC standard after each 10 sample injections.

#### 4.2.3. HPLC-MS Analysis

LC-MS experiments were carried out at the Institute of Biomedical Chemistry using equipment of “Human proteome” Core Facility Center (Moscow, Russia). For samples obtained after continuous cultivation, HPLC-MS analysis was performed using liquid chromatography system Ultimate 3000 Nano LC (Thermo Fisher Scientific, Waltham, MA, USA). The HPLC system was coupled to the Q Exactive Hybrid Quadrupole-Orbitrap mass spectrometer with ESI (Thermo Fisher Scientific, Waltham, MA, USA). The HPLC-MS method for the analysis was as follows: Zorbax SB-C18 column (150 × 0.5 mm, 5 µm, Agilent, Santa Clara, CA, USA), injection volume 20 µL in three replicates per sample, and column temperature 40 °C. The mobile phase A consisted of the aqueous solution of 0.1% formic acid, and the eluent B was water-acetonitrile-formic acid (20/80/0.1, *v*/*v*/*v*) mixture. The gradient program was the following: 0 min—5% B, 20 min—35% B, 25 min—99% B, 30 min—99% B, 30.5 min—5% B, 35 min—5% B. The flow rate was 0.2 mL/min. The mass spectrometer was operated in the positive-ion mode under the following conditions: mass range of *m*/*z* 100–1500, with a resolution of 60,000.

For samples obtained after discontinuous cultivation, HPLC-MS analyses were performed using liquid chromatography system Ultimate 3000 Nano LC (Thermo Fisher Scientific, Waltham, MA USA) coupled to the hybrid quadrupole time-of-flight Maxis Impact Q-ToF mass spectrometer with ESI (Bruker Daltoniks, Germany). The HPLC-MS method for the analysis was as follows: Zorbax SB-C18 column (150 × 0.5 mm, 5 µm, Agilent, Santa Clara, CA, USA), injection volume 20 µL in three replicates per sample, and column temperature 40 °C. The mobile phase A consisted of an aqueous solution of 0.1% formic acid, and the eluent B was 0.1% formic acid in acetonitrile. The gradient program was the following: 0 min—5% B, 15 min—5% B, 25 min—95% B, 30 min—95% B, 31 min—5% B, 34 min—5% B The flow rate was 0.04 mL/min. The mass spectrometer was operated in the positive-ion mode under the following conditions: mass range of *m*/*z* 100–1500, with a resolution of 50,000, capillary voltage 4.1 kV, nebulizer gas 0.4 Bar, dry gas 4.0 L/min, dry gas temperature 180 °C.

### 4.3. Data Processing and Statistical Analysis

Peak detection, noise signal removal, data processing, and analysis were carried out by XCMS package on Workflow4Metabolomics (www.workflow4metabolomics.org accessed on 8 January 2021) web platform [75] with the following parameters: peak detection algorithm was centwave; max tolerated *m*/*z* deviation was 10 ppm for Orbitrap data and 15 ppm for qToF data; minimum and maximum peak width was 5 s and 50 s respectively.

Partial least square discriminant analysis (PLS-DA) [76] and Orthogonal partial least square analysis (OPLS-DA) [77] were used for the multivariate statistics in multigroup and pairwise comparisons, respectively. *t*-test was used for pairwise univariate statistics. The significance threshold was *p*-value < 0.05. Data normalization and statistical analysis were performed by the Metaboanalyst web platform [78].

## 5. Patients

Retrospective study included patients (*n* = 21 and *n* = 12 for continuous and discontinuous protocols respectively), undergoing IVF programs from March 2017 to October 2019 in the Department of Assisted Reproductive Technologies of the National Medical Research Center of Obstetrics, Gynecology, and Perinatology named after Academician V.I.Kulakov (Ministry of Health of the Russian Federation). The study was approved by the Ethics Committee of the National Medical Research Center for Obstetrics, Gynecology, and Perinatology named after Academician V.I.Kulakov (Protocol 2 from 9 February 2017). All study participants provided written informed consent.

Inclusion criteria were age 24–37 years old, body mass index 18–29 kg/m^2^, primary infertile women with major indications for IVF, tuboperitoneal infertility, more than 5 oocytes were collected during IVF, the couples had declared no history of chromosomal abnormalities. Exclusion criteria were low ovarian reserve, uterus malformations, abnormal endometrial development, endometriosis, gynecological tumors, a history of recurrent pregnancy loss, any somatic or mental disorders contradictory for pregnancy and childbirth, any acute inflammatory processes, acute exacerbation of any chronic disease, pathozoospermia. Patients with no follicular growth effect after stimulation or unsuccessful oocyte retrieval were excluded as well.

## Figures and Tables

**Table 1:** Distribution of 5-day embryos into arbitrary classes. Alphanumerical morphological grades for blastocysts in Classes 1–3 were evaluated based on Gardner classification. The numbers of samples in each group and the distribution of euploid/aneuploid embryos are shown as well.

Groups	Class 1		Class 2		Class 3	Class 4
	“Excellent”		“Good”		“Fair”	“Poor”
Grades	6AA, 6BA, 4AA, 4AB, 3AA		4BA, 4BB, 3AB, 3BB, 3BA		3BC, 3CB, 3CC	10b-5b, 10c-3c, 2pb, 2pn, atresia
Karyotype	Euploid	Aneuploid	Euploid	Aneuploid	N/D	N/D
Number	23	12	22	21	20	55

**Table 2:** Molecular ions which distinguish day 5 culture media of aneuploid and euploid embryos. RT—retention time; FC—Fold Change (significantly different between groups with *p* < 0.05 based on univariate statistics). Italics indicate molecular ions identified by HMDB: *m*/*z* 309.204—16-hydroxy-10-oxohexadecanoic acid; *m*/*z* 389.252—5,6-dihydroxyprostaglandin F1.

*m*/*z*	Retention Time	Fold Change	*m*/*z*	Retention Time	Fold Change
314.780	0.94	2.42	354.896	1.02	3.28
450.755	0.96	2.29	330.934	1.05	2.31
448.789	0.97	2.03	*309.204*	1.34	2.76
436.871	0.97	2.64	370.281	11.98	3.11
333.844	0.97	2.97	248.106	13.6	2.31
411.860	0.97	4.72	354.286	14.58	2.03
234.171	0.98	3.25	412.328	16.64	2.06
530.769	0.99	2.64	*389.252*	16.65	3.11
316.87	0.99	2.04	384.296	16.66	−2.00
410.857	0.99	2.52	368.273	16.67	2.79
402.825	1.00	2.66	169.978	29.8	2.02

**Table 3:** Pathway enrichment of molecular ion identifications contributing to the difference in day 5 SECM of aneuploid and euploid embryos. Molecular ions filtered by PLS-DA were mapped to HMDB database, list of identified metabolites was then mapped to KEGG pathways database. ‘p’ refers to the raw *p*-value determined by Fisher’s exact test; ‘Impact’ refers to topological impact on pathway (routing betweenness centrality score).

Pathway Name	Matched	*p*	Impact
Pentose phosphate pathway	5/22	0.51	0.48
Valine, leucine, and isoleucine biosynthesis	2/8	0.53	0
Vitamin B6 metabolism	2/9	0.6	0.57
Phenylalanine metabolism	2/10	0.66	0.14
alpha-Linolenic acid metabolism	2/13	0.8	0
Butanoate metabolism	2/15	0.86	0.03
Histidine metabolism	2/16	0.88	0.28
Retinol metabolism	2/17	0.9	0.22
Caffeine metabolism	1/10	0.91	0
Biotin metabolism	1/10	0.91	0.2
Terpenoid backbone biosynthesis	2/18	0.92	0.01
Pentose and glucuronate interconversions	2/18	0.92	0.08
Arginine and proline metabolism	5/38	0.93	0.26
Pantothenate and CoA biosynthesis	2/19	0.93	0.01
Glycine, serine, and threonine metabolism	4/33	0.94	0.15
Valine, leucine, and isoleucine degradation	5/40	0.95	0.03
Alanine, aspartate, and glutamate metabolism	3/28	0.95	0.09

**Table 4:** Selected molecular ions identified in SECM from implanted embryos after 5 days of continuous cultivation. Identifications were performed by mapping molecular ions *m*/*z* to HMDB metabolites database (accuracy ≤ 15 ppm). Fold change indicates relative change of mean integrated area of chromatographic peak for this ion when compared with SECM from embryos with unsuccessful implantation.

*m*/*z*	Retention Time	Name	Fold Change
110.058	1.04	4-Aminobutyraldehyde	−3.03
114.066	1.12	Creatinine	−2.42
116.071	1.06	L-Proline	−2.27
118.086	1.06	L-Valine	−5.8
119.082	1.35	L-2,4-diaminobutyric acid	−2.09
130.087	1.00	Pipecolic acid	−2.9
132.077	1.55	Creatine	−2.91
147.066	8.1	Adipic acid	−7.47
153.055	1.03	p-Hydroxyphenylacetic acid	−2.14
163.072	1.00	L-4-Hydroxyglutamine	−5.04
166.087	2.58	L-Phenylalanine	−88.24
219.111	7.83	N-Acetylserotonin	−15.24
227.08	7.62	L-Tryptophan	−2.7
264.106	9.68	L-erythro-tetrahydrobiopterin	−3.32
269.09	13.67	Inosine	−2.01
280.093	1.05	Glycerophosphocholine	−2.28
309.204	1.33	Hexadecanedioic acid	−2.61
469.051	1.04	CDP-ethanolamine	−2.36

**Table 5:** Pathway enrichment of molecular ions identifications contributing to the difference in SECM from embryos with successful and unsuccessful implantation in continuous protocol. Molecular ions filtered by PLS-DA were mapped to HMDB database, list of identified metabolites was then mapped to KEGG pathways database. ‘p’ refers to the raw *p*-value determined by Fisher’s exact test; ‘Impact’ refers to topological impact on pathway (routing betweenness centrality score).

Pathway Name	Matched	*p*	Impact
Vitamins			
Vitamin B6 metabolism	2/9	0.48	0.13
Lipids			
Linoleic acid metabolism	3/5	0.04	0
Steroid biosynthesis	5/42	0.88	0.09
Amino acids			
Valine, leucine and isoleucine biosynthesis	2/8	0.42	0
Valine, leucine and isoleucine degradation	4/40	0.94	0.04
Phenylalanine metabolism	2/10	0.54	0.36
Histidine metabolism	2/16	0.8	0.22
Arginine and proline metabolism	5/38	0.82	0.1
Cysteine and methionine metabolism	4/33	0.86	0.14
Tyrosine metabolism	5/42	0.88	0.12
Aminoacyl-tRNA biosynthesis	5/48	0.94	0
Other			
Butanoate metabolism	2/15	0.77	0.14
Propanoate metabolism	2/23	0.93	0.04
Caffeine metabolism	3/10	0.25	0.69
Pentose and glucuronate interconversions	4/18	0.39	0.2
Nicotinate and nicotinamide metabolism	1/15	0.94	0

**Table 6:** Pathway enrichment of molecular ions identifications contributing to the difference in 3rd day SECM from embryos with successful and unsuccessful implantation in discontinuous protocol. Molecular ions filtered by PLS-DA were mapped to HMDB database, list of identified metabolites was then mapped to KEGG pathways database. ‘p’ refers to the raw *p*-value determined by Fisher’s exact test; ‘Impact’ refers to topological impact on pathway (routing betweenness centrality score).

Pathway Name	Matched	*p*	Impact
Vitamins			
Vitamin B6 metabolism	2/9	0.43	0.54
Retinol metabolism	5/17	0.11	0.22
Lipids			
alpha-Linolenic acid metabolism	3/13	0.34	0.33
Glycerophospholipid metabolism	3/36	0.94	0.12
Biosynthesis of unsaturated fatty acids	4/36	0.84	0.00
Terpenoid backbone biosynthesis	1/18	0.96	0.00
Sphingolipid metabolism	1/21	0.97	0.00
Amino Acids			
Histidine metabolism	1/16	0.94	0.19
Arginine biosynthesis	1/14	0.91	0.08
Arginine and proline metabolism	3/38	0.95	0.14
Cysteine and methionine metabolism	2/33	0.98	0.06
Valine, leucine, and isoleucine degradation	3/40	0.96	0.01
Valine, leucine, and isoleucine biosynthesis	3/8	0.12	0.00
Sugars			
Pentose and glucuronate interconversions	1/18	0.96	0.14
Starch and sucrose metabolism	1/18	0.96	0.00
Other			
Caffeine metabolism	2/10	0.48	0.00

**Table 7:** Pathway enrichment of molecular ions identifications contributing to the difference in 5th day SECM from embryos with successful and unsuccessful implantation in discontinuous protocol. Molecular ions filtered by PLS-DA were mapped to HMDB database, list of identified metabolites was then mapped to KEGG pathways database. ‘p’ refers to the raw *p*-value determined by Fisher’s exact test; ‘Impact’ refers to topological impact on pathway (routing betweenness centrality score).

Pathway Name	Matched	*p*	Impact
Vitamins			
Vitamin B6 metabolism	2/9	0.63	0.54
Retinol metabolism	3/17	0.77	0.37
Amino acids			
Phenylalanine metabolism	4/10	0.17	0.74
Phenylalanine, tyrosine, and tryptophan biosynthesis	2/4	0.22	1.00
Valine, leucine, and isoleucine, biosynthesis	3/8	0.26	0.00
Arginine biosynthesis	2/14	0.86	0.08
Arginine and proline metabolism	6/38	0.89	0.11
Lipids			
Arachidonic acid metabolism	10/36	0.28	0.26
Terpenoid backbone biosynthesis	3/18	0.81	0.19
Sugars			
Galactose metabolism	5/27	0.76	0.12
Other			
Ubiquinone and other terpenoid-quinone biosynthesis	2/9	0.63	0.00
Caffeine metabolism	5/10	0.05	1.00

## Data Availability

Data is contained within the article or Supplementary Materials.

## Supplementary Materials

The supporting information can be downloaded at: https://www.mdpi.com/article/10.3390/ijms23052706/s1.
