# Supplementary material for: LC-MS Analysis Revealed the Significantly Different Metabolic Profiles in Spent Culture Media of Human Embryos with Distinct Morphology, Karyotype and Implantation Outcomes
Source: Int J Mol Sci. 2022 Feb 28;23(5):2706. doi: 10.3390/ijms23052706 (PMC8911215; doi:10.3390/ijms23052706)
Supplement: Supplementary file 1 [file ijms-23-02706-s001.zip › Table S3.pdf]

List of tested amino-acids(Labeled standard or ms-ms-analysis)

#	Name	Formula	m/z[M+H]	m/z[M+H]Labeled std.
1	L-lysine	C6H14N2O2	147.1128	155.127
2	L-histidine	C6H9N3O2	156.076	165.087
3	L-Arginine	C6H14N402	175.118	185.127
4	L-Tyrosine	C9H11NO3	182.0811	192.1083
5	L-Phenylalanine	C9H11NO2	166.086	176.113
6	L-methionine	C5H11NO2S	150.0583	156.072
7	L-leucine	C6H13NO2	132.1019	139.119
8	L-isoleucine	C6H13NO2	132.1019	139.119
9	L-valine	C5H11NO2	118.086	124.1
10	L-proline	C5H9NO2	116.0706	122.0844
11	L-Glutamine	C5H10N2O3	147.0764	
12	L-Tryptophan	C11H12N2O2	205.0977	
