# Supplementary figures and images for: LC-MS Analysis Revealed the Significantly Different Metabolic Profiles in Spent Culture Media of Human Embryos with Distinct Morphology, Karyotype and Implantation Outcomes
Source: Int J Mol Sci. 2022 Feb 28;23(5):2706. doi: 10.3390/ijms23052706 (PMC8911215; doi:10.3390/ijms23052706)

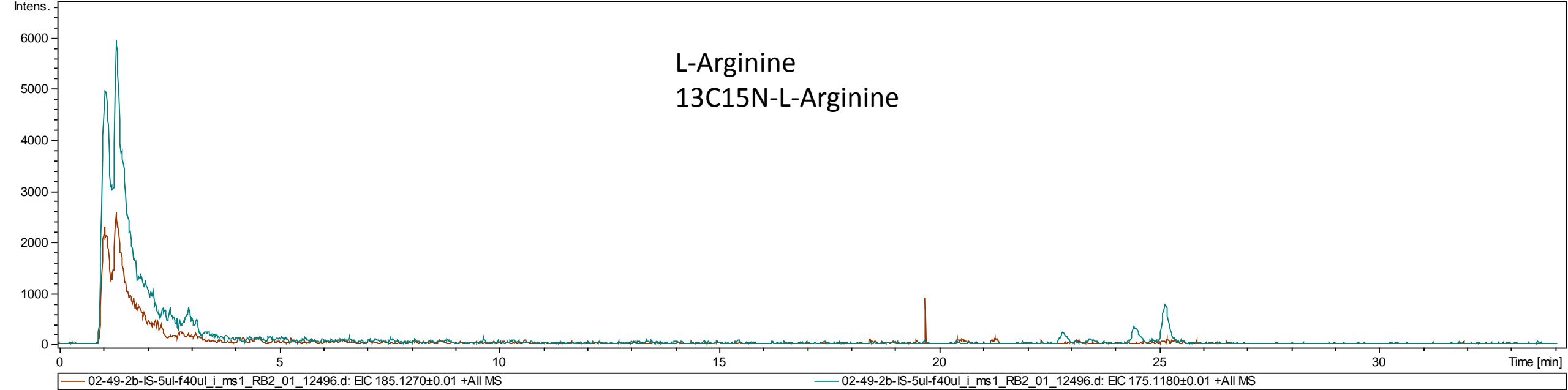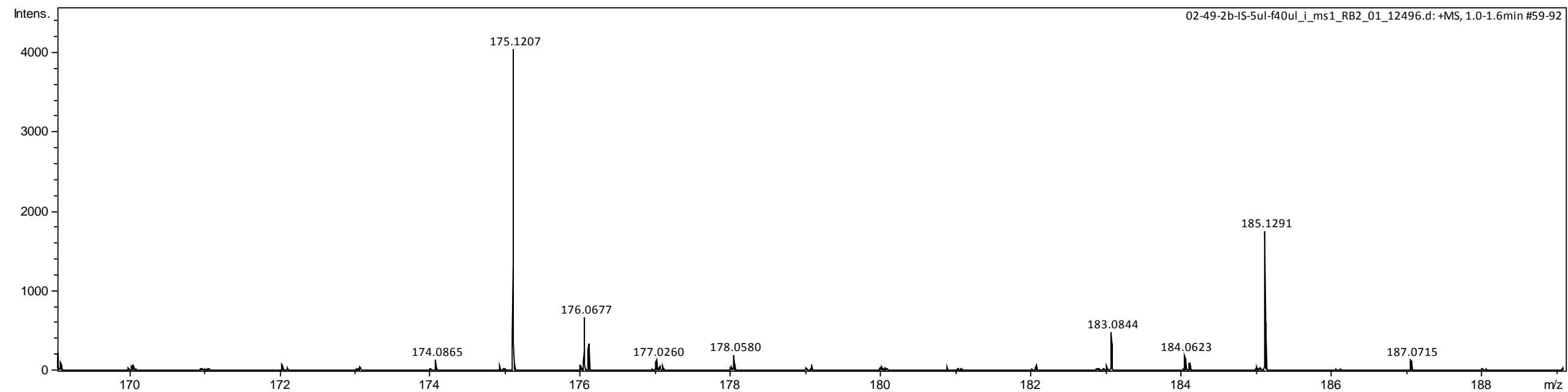

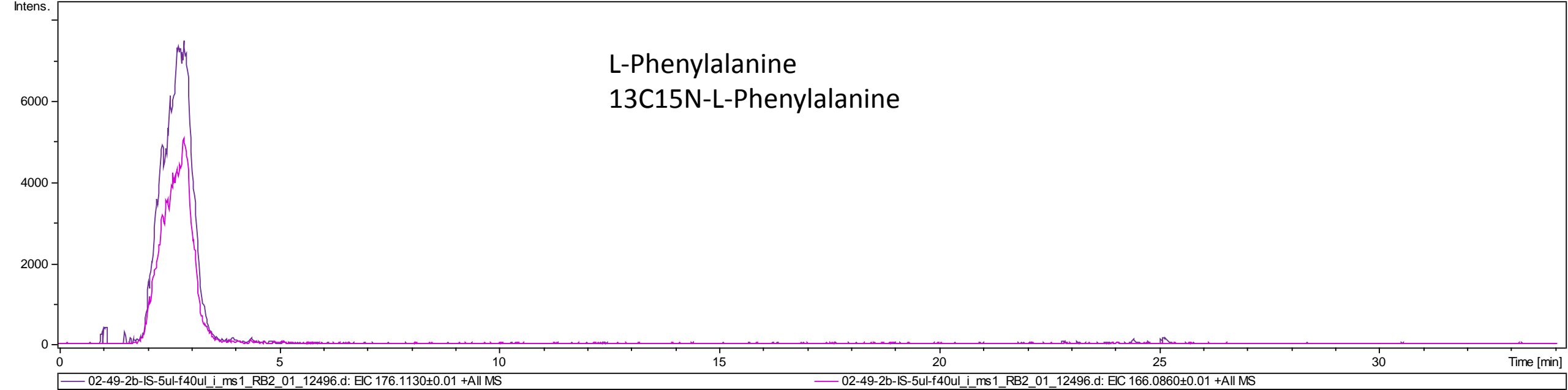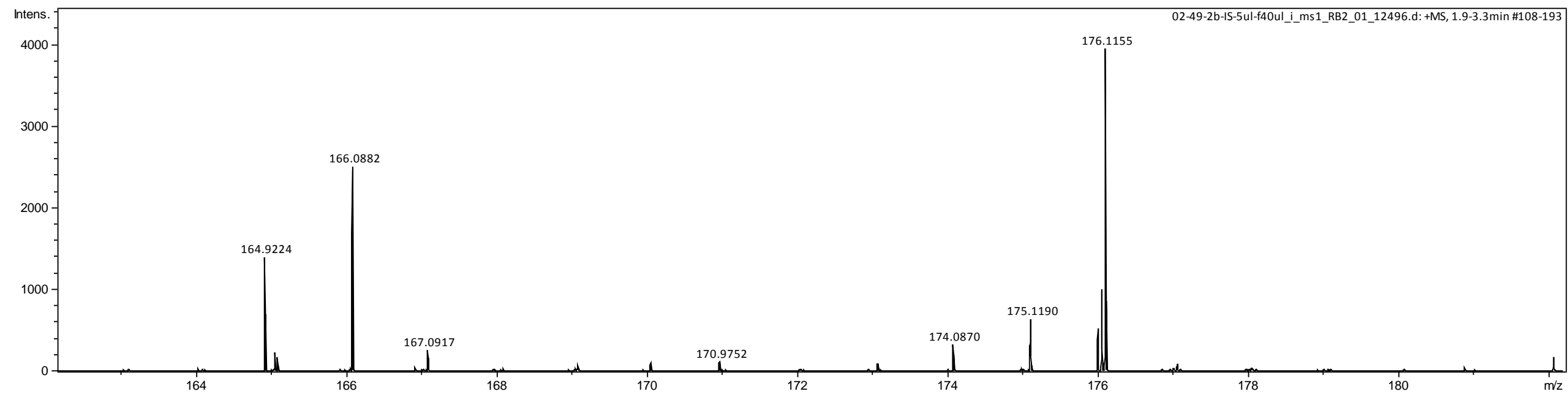

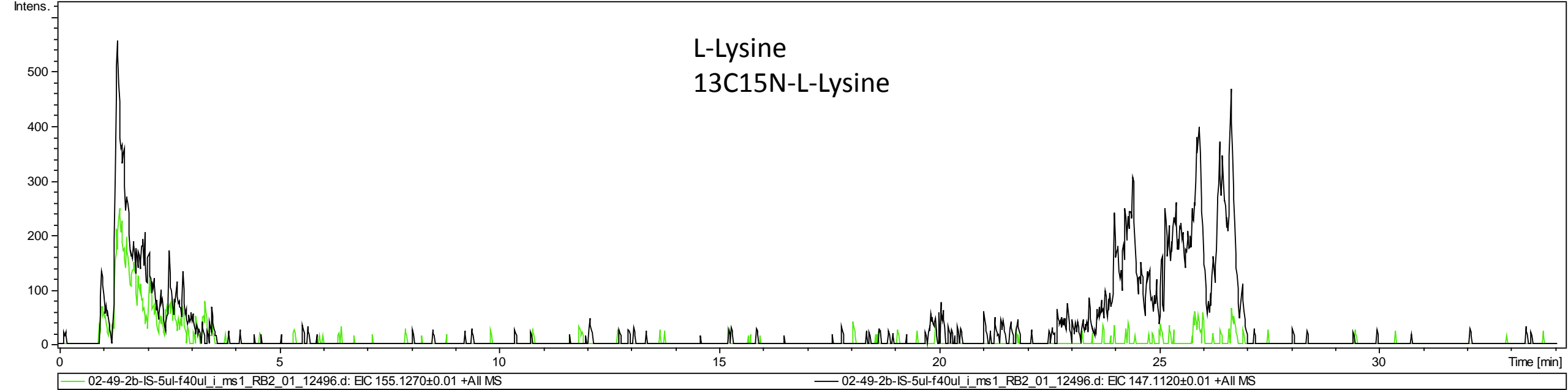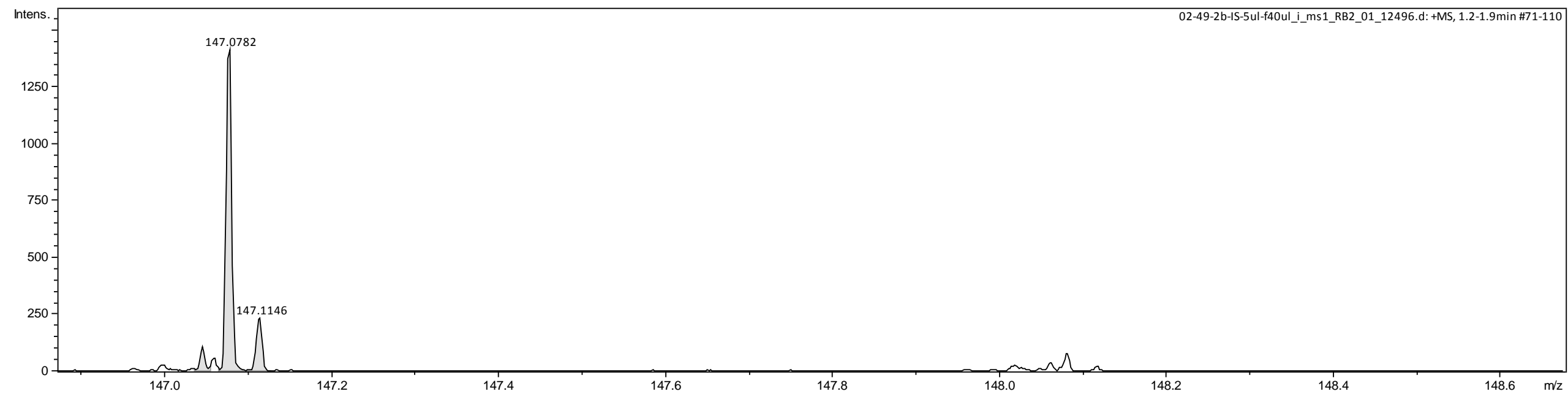

L-Histidine  
<sup>13</sup>C<sup>15</sup>N-L-Histidine

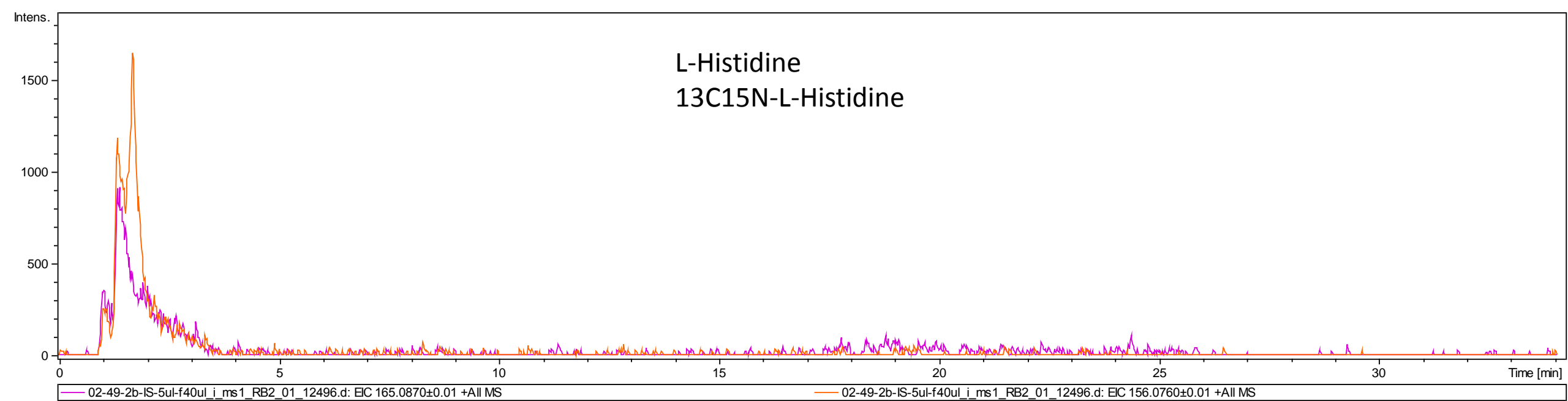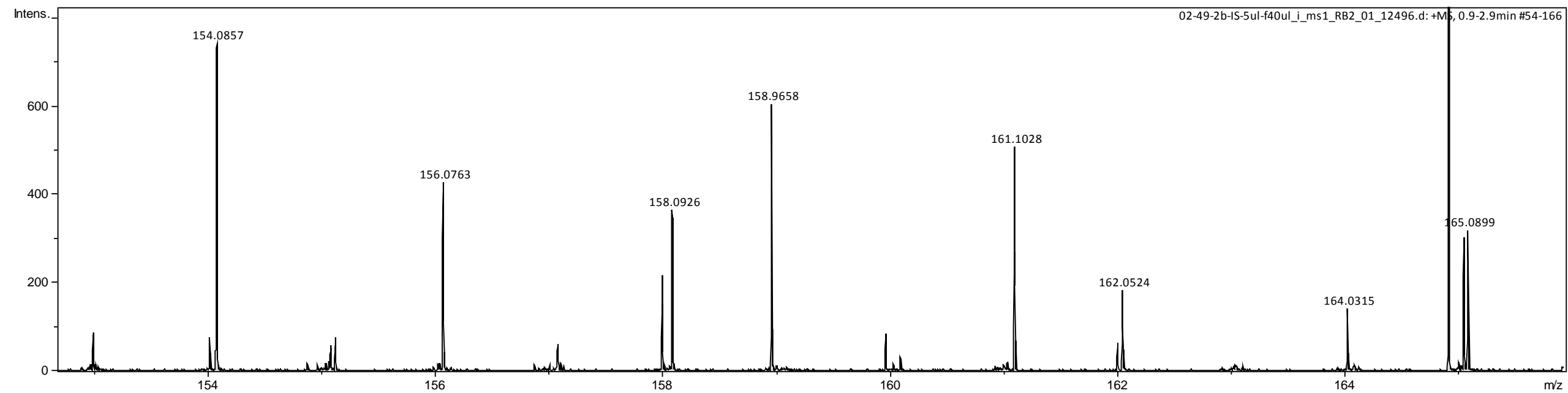

L-Proline  
13C15N-L-Proline

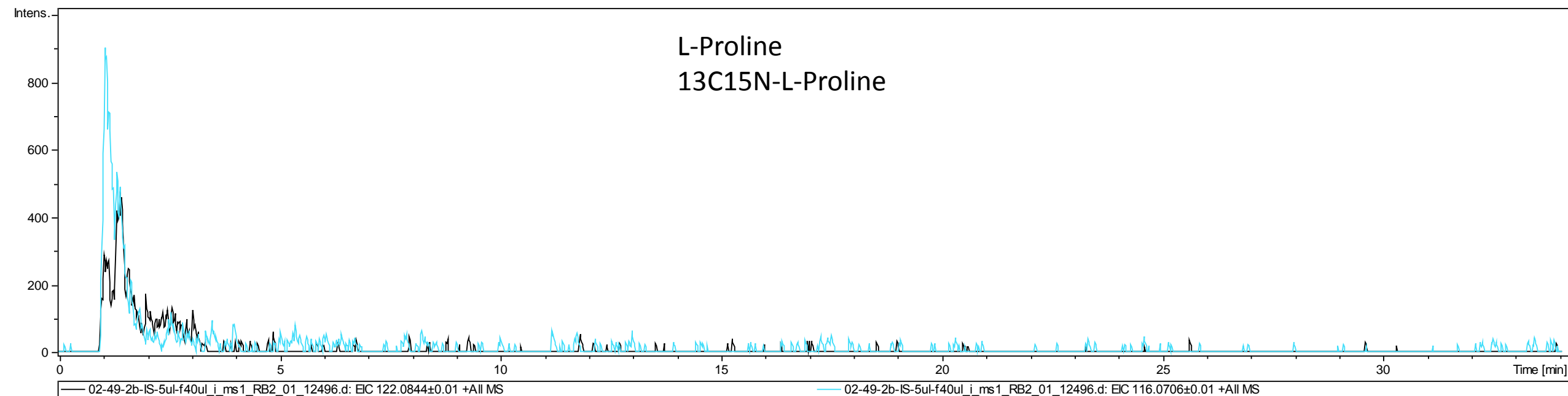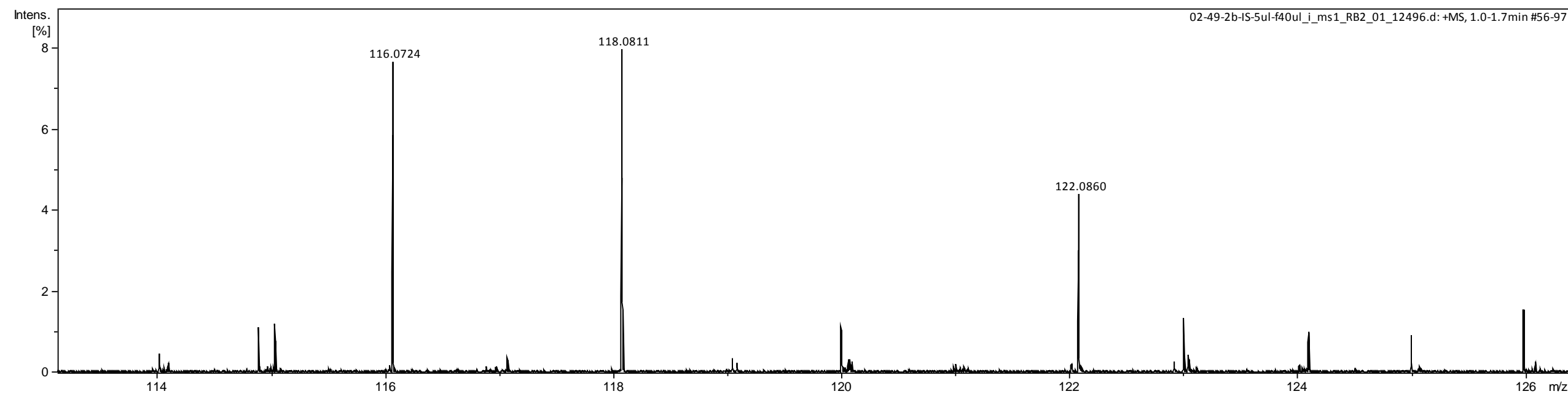

L-Valine  
13C15N-L-Valine

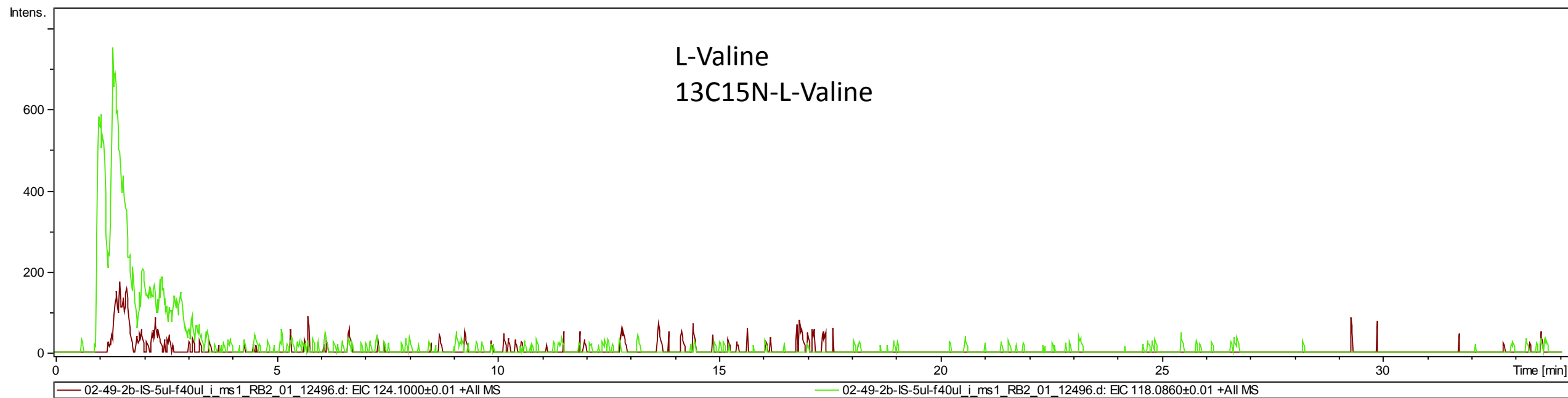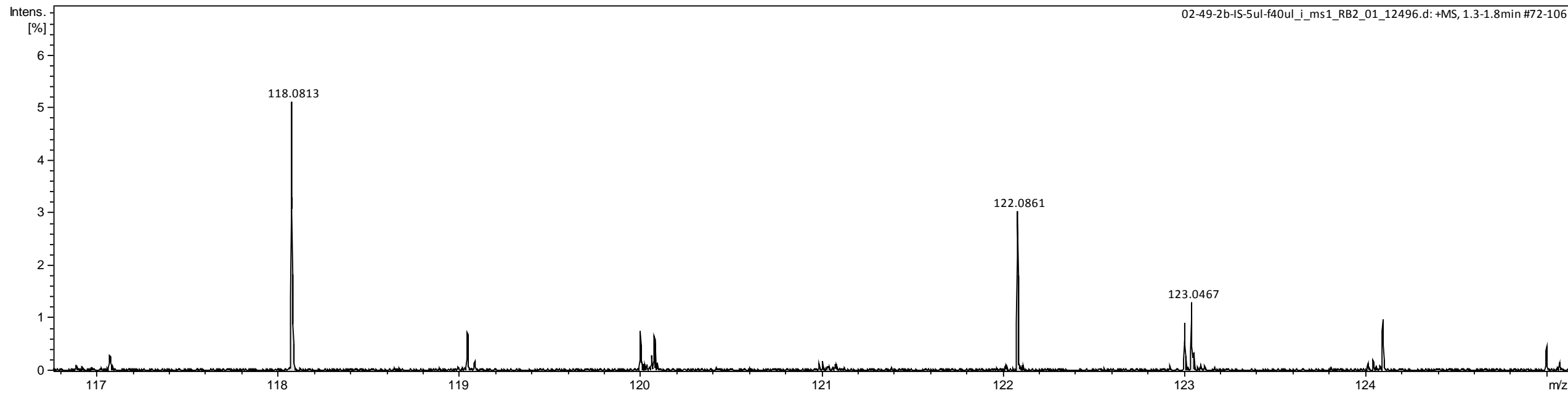

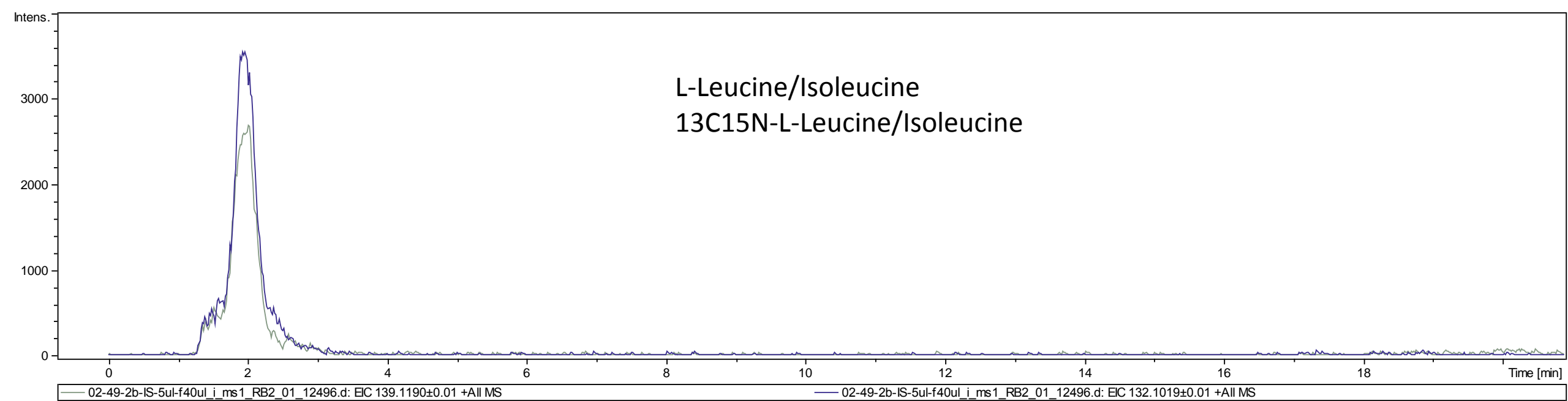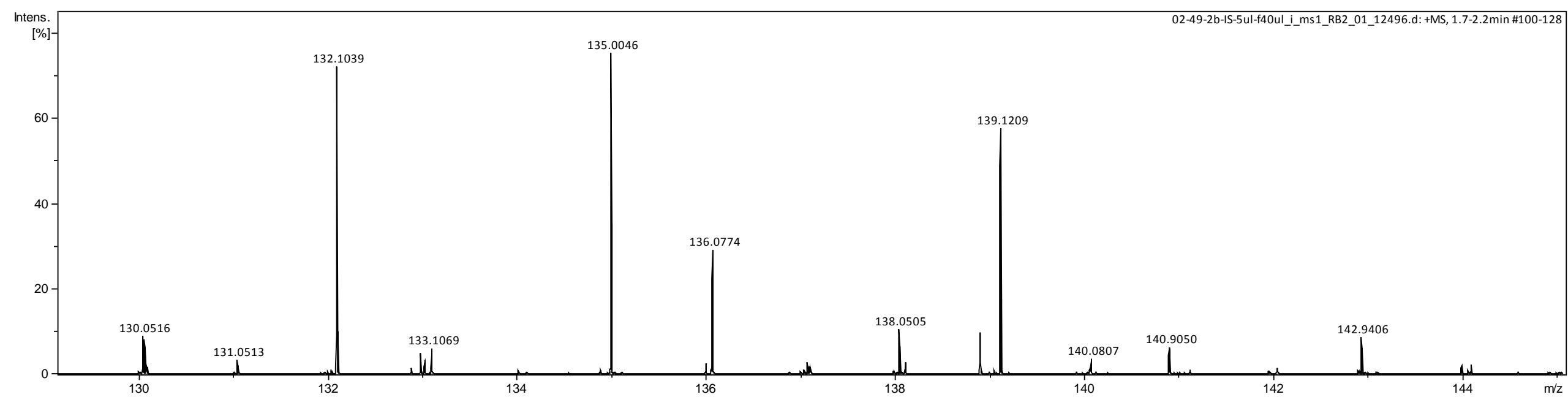

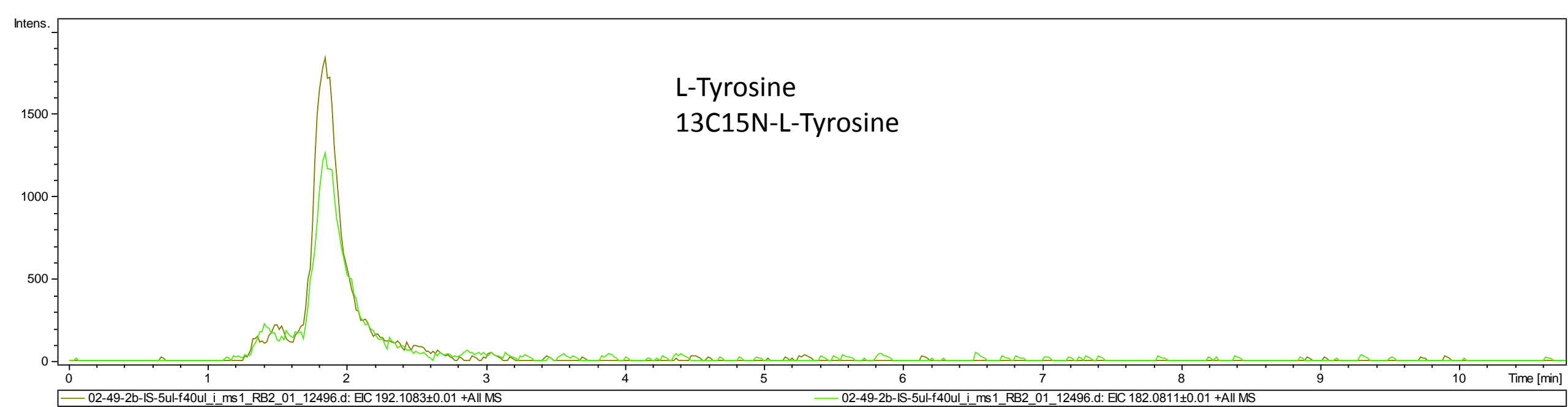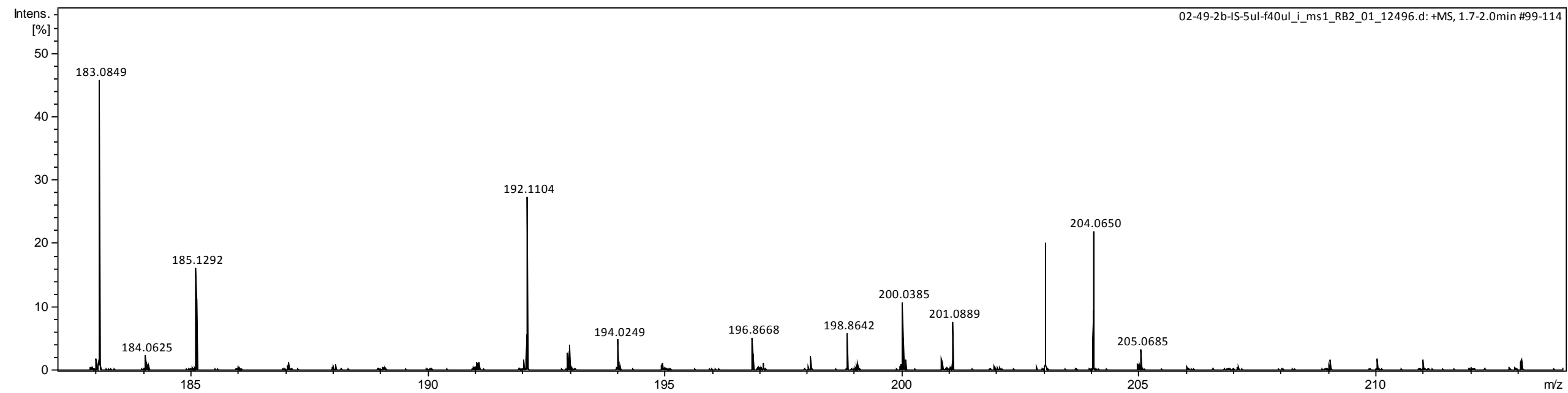

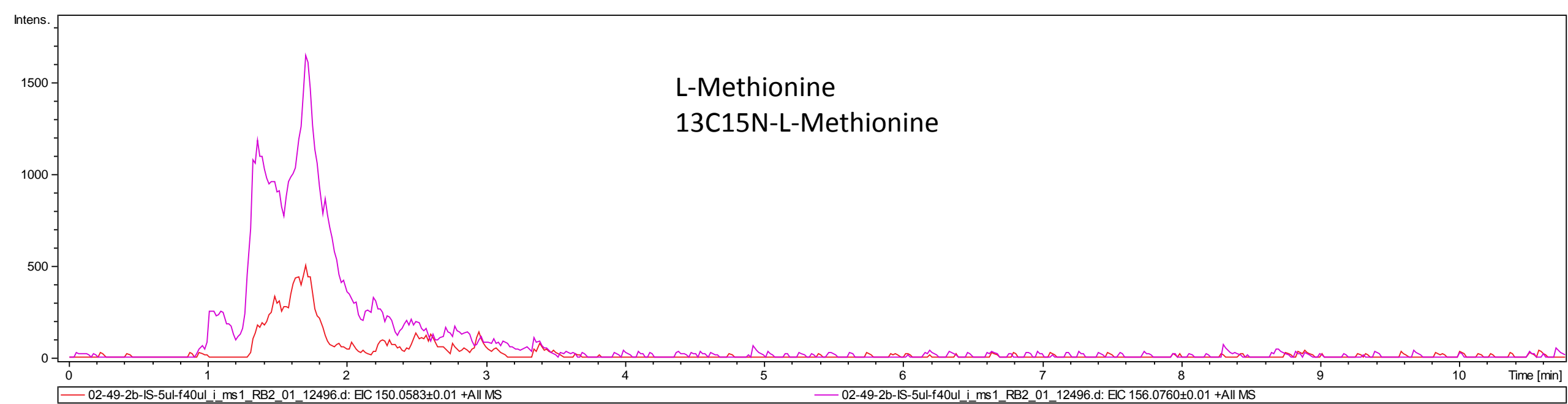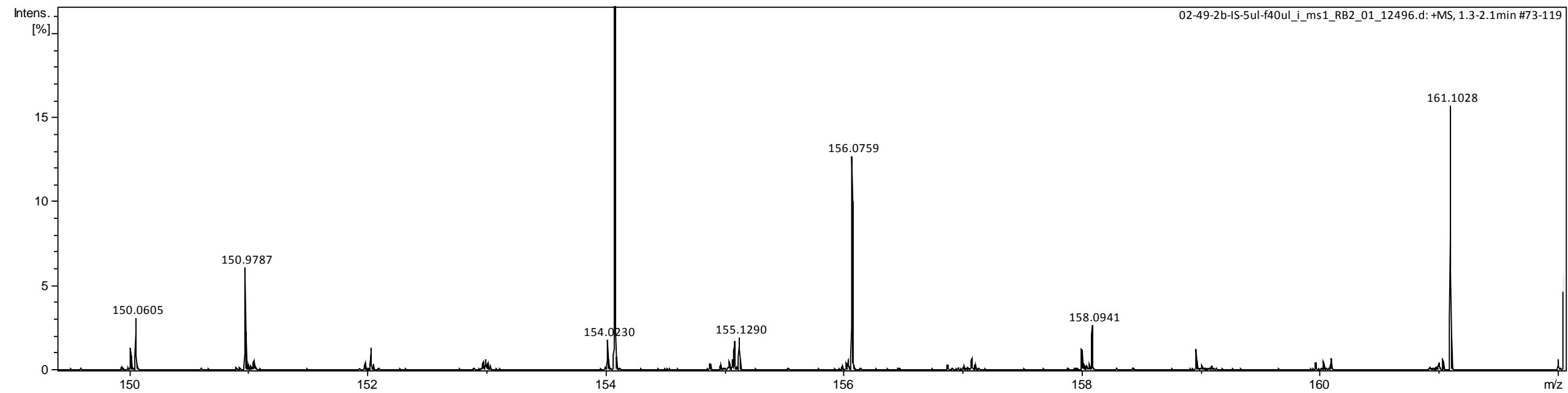

# L-Glutamine

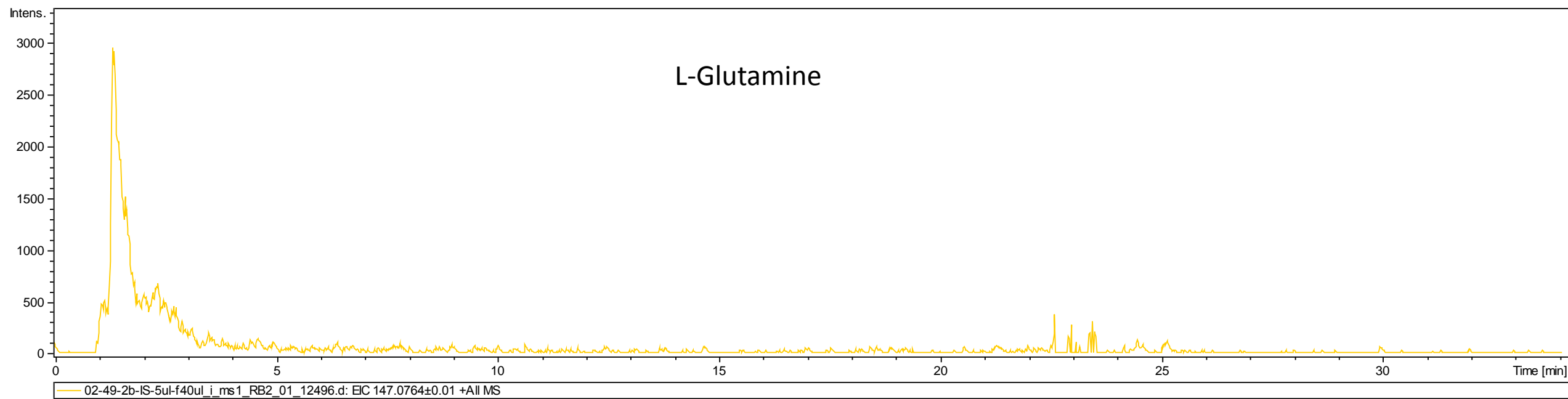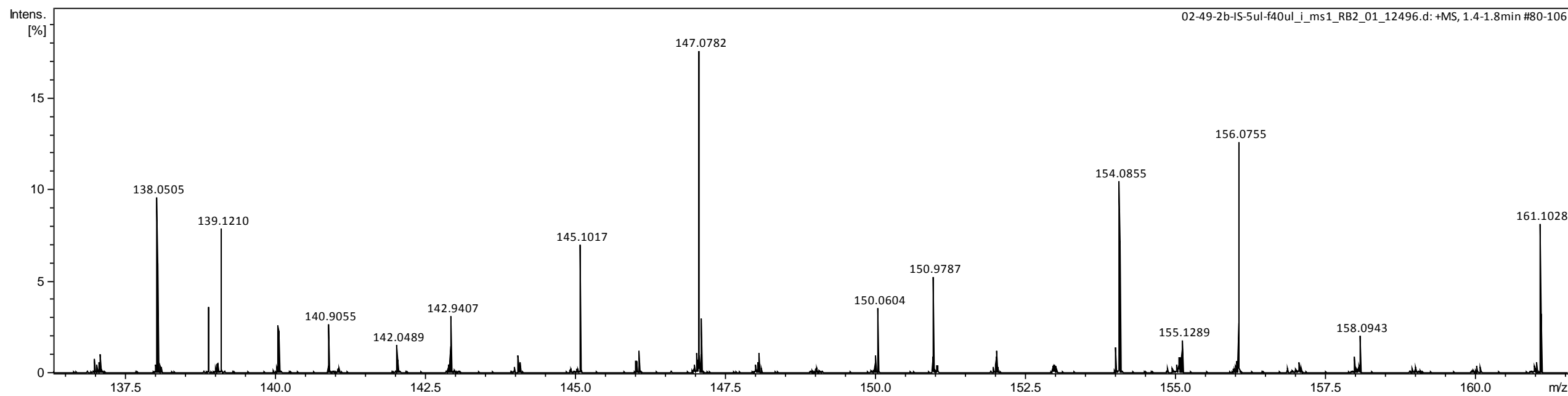

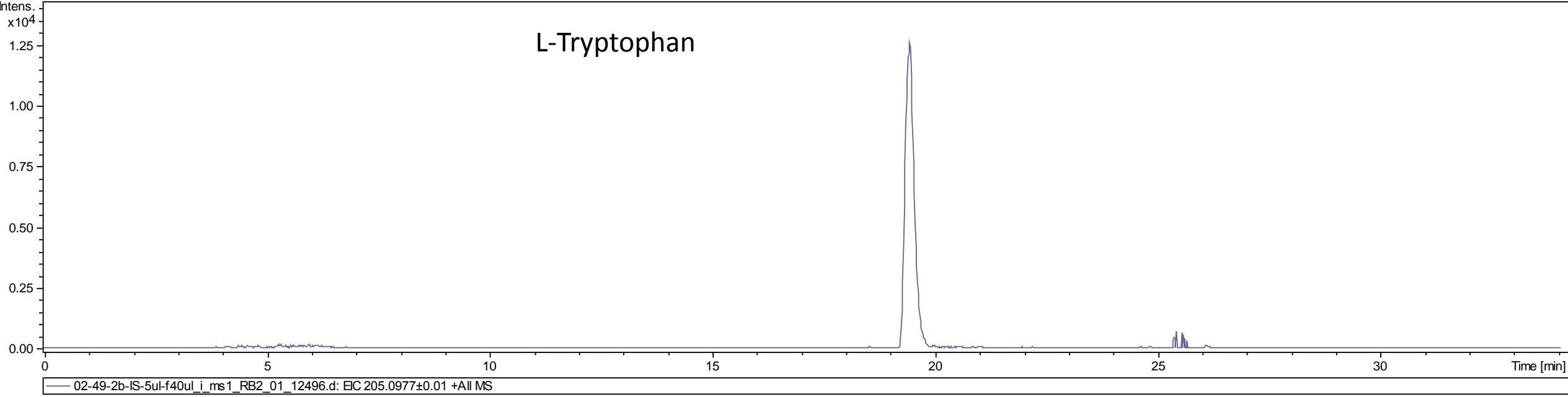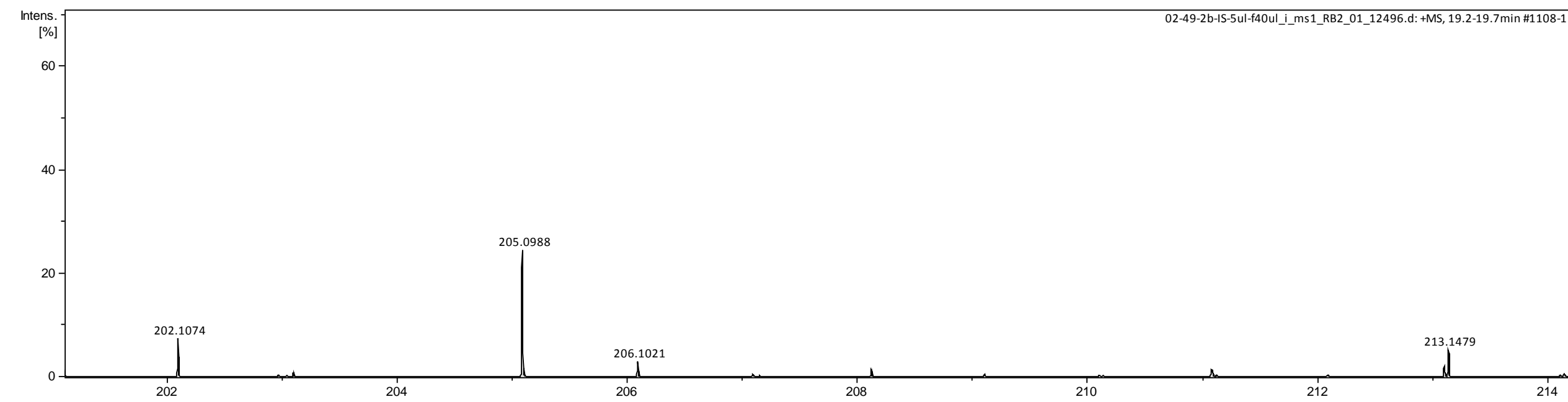

Supplement: Supplementary file 1 [file ijms-23-02706-s001.zip › Figure S1.pdf]
